# Harnessing artificial intelligence for breakthroughs in lung cancer management: are we ready for the future?

**DOI:** 10.3389/fonc.2024.1450568

**Published:** 2024-09-20

**Authors:** Luca Bertolaccini, Juliana Guarize, Cristina Diotti, Stefano Maria Donghi, Monica Casiraghi, Antonio Mazzella, Lorenzo Spaggiari

**Affiliations:** ^1^ Department of Thoracic Surgery, IEO, European Institute of Oncology IRCCS, Milan, Italy; ^2^ Division of Interventional Pulmonology, IEO, European Institute of Oncology IRCCS, Milan, Italy; ^3^ Department of Oncology and Hemato-Oncology, University of Milan, Milan, Italy

**Keywords:** lung cancer, artificial intelligence, artificial neural network, preoperating management, minimally invasive therapy

## Introduction

Artificial Intelligence (AI) is a broad field encompassing the science and engineering of creating intelligent machines and brilliant computer programs. The essence of AI involves the ability of machines to perform tasks that would typically require human intelligence. These tasks include reasoning, learning, problem-solving, perception, language understanding, and interaction. Despite numerous attempts, only some universally accepted definitions of intelligence relate to human-like characteristics. The challenge remains to define the computational procedures that constitute intelligent behaviour broadly and consistently. AI can be broadly categorised into several types. Narrow AI (weak AI) is designed for a specific function, such as video games, personal assistants, or recommendation engines. General AI (strong AI) is a sophisticated system that can perform human-like tasks, such as self-driving cars or hospital operating rooms. Lastly, super AI is a theoretical concept representing a system that surpasses human cognitive abilities in thinking, reasoning, and learning. AI systems typically involve algorithms that enable them to learn from data and adapt to new situations. Critical components of AI include machine learning, deep learning, and natural language processing, which are used in various applications such as computer vision, speech recognition, and expert systems (1). AI is also not a monolithic field but encompasses multiple perspectives and methodologies. The Human-like AI focuses on simulating human intellect and behaviour on machines; it includes efforts to model common-sense reasoning, expert knowledge in law and medicine, and social behaviour.

On the other hand, rational-like AI emphasises the ability of machines to achieve goals based on performance measures; it involves creating systems that act rationally to achieve specified outcomes (2). AI can amplify human capabilities by analysing vast volumes of data quickly and accurately, freeing up work that requires uniquely human skills like professional judgment, creativity, and customer empathy. A good data strategy is essential for managing legal data, involving recognition, storage, accessibility, and publication, to ensure effective use and management (3).

## History of artificial intelligence

The definition of AI is a term coined by John McCarthy in 1955, which refers to the science and engineering of making intelligent machines (4). The history of AI spans several decades, with significant milestones and developments that have shaped the field. Between 1943 and 1952, AI transitioned from a concept to tangible experiments and practical applications. From 1952 to 1956, AI surfaced as a unique domain of investigation. This period saw the groundwork laid for the revolutionary technological domain that AI would become. The period from 1956 to 1974 is known as the *Golden Age* of AI. Researchers and innovators achieved remarkable advancements in the field, driven by their enthusiasm for AI. In 1974, a critical report on AI research led to significant funding cuts and a sense of letdown. This period is known as the First AI Winter. Between 1980 and 1987, AI underwent a renaissance, with renewed interest and advancements in the field. The period from 1987 to 1993 saw another decline in AI research funding and interest, known as the Second AI Winter. Between 1993 and 2011, AI professionals shifted from attempting to match human intelligence to crafting pragmatic, ingenious software tailored to specific tasks (5).

## Real-world applications of artificial intelligence

AI will profoundly impact various industries, including healthcare, finance, and transportation (6). AI endeavours to think humanly, engaging in introspection and solving general problems akin to cognitive sciences. It also strives to reason, using logic to represent and reason within domains and to act rationally, where agents perceive and act upon their environments. However, practical issues arise in representing and reasoning within complex domains and acting rationally or designing agents that perceive their environment and take actions to achieve their goals, illustrated by scenarios like the Prisoner’s Dilemma, which exemplifies the conflict that can occur between the interests of individuals and the interests of the collective (7).

Generative AI tools can generate text in multiple languages, interpret images, and connect to various services and data through plugins. These tools, including Midjourney for image generation, exemplify AI’s integration into everyday software as task assistants. Large Language Models like ChatGPT (Generative Pre-trained Transformer) allow natural and conversational interactions with computers. Other notable Large Language Models include Bard, Gemini, and Claude. Despite their advanced capabilities, Large Language Models have limitations. They are highly trained text predictors, and their responses, based on probable language patterns, may include plausible yet incorrect information. Large Language Models lack access to current data, cannot perform complex computations, and do not interpret language beyond their training data. Large Language Models also have ethical concerns, such as privacy issues, cognitive biases, environmental impacts due to significant carbon emissions during training, and labour exploitation. The aim is to ensure AI’s safety and compliance with fundamental rights. It imposes safeguards on general-purpose AI and limits biometric identification. The act addresses privacy concerns, intellectual property violations, and the need for transparency in AI development and deployment (8). The real-world applications of AI are diverse and widespread, impacting various sectors and industries (9).

The use of AI in surgery raises questions about the extent of machine autonomy and the potential for diminished human oversight. Issues such as informed consent, patient autonomy, and the transparency of AI decision-making processes need to be carefully considered. Moreover, the potential for AI to perpetuate or exacerbate existing biases in healthcare decisions requires vigilant oversight and the development of fair algorithms. The introduction of AI in surgery also presents significant legal challenges, particularly regarding liability. Determining accountability in an AI-related surgical error is complex, mainly when AI systems operate with a high degree of autonomy. Legal frameworks must evolve to address these challenges, ensuring that the use of AI in surgery does not compromise patient safety or legal protections (10).

## Artificial intelligence in lung cancer management

### Artificial intelligence in lung cancer oncology

AI has been increasingly applied in lung cancer, potentially improving patient outcomes (**Table 1**). AI algorithms like machine learning, deep learning, and radiomics have shown remarkable capabilities in detecting and characterising lung nodules, aiding in accurate lung cancer screening and diagnosis. AI models can integrate multiple imaging modalities, such as low-dose CT scans, PET-CT imaging, and chest radiographs, to provide a more comprehensive diagnostic assessment. AI models can utilise biomarkers and tumour markers as supplementary screening tools, enhancing the specificity and accuracy of early detection. AI models can predict treatment responses and guide the selection of optimal therapies, improving patient outcomes. AI algorithms can optimise radiation therapy for lung cancer patients, enhancing treatment efficacy and reducing side effects (11–13). AI-driven applications in the management of NSCLC, with a specific emphasis on radiomics and pathomics. It also critically examines the current limitations and prospects in this area. The thoracic cancer community should be undeterred by the anticipated lengthy process of integrating AI into daily clinical practice, as its profound influence on personalised treatment methods is indisputable (14).

**Table 1:** Applications of Artificial Intelligence (AI) in Lung Cancer Management.

Application Area	AI Techniques Used	Description
Screening and Diagnosis	Machine Learning, Deep Learning, Radiomics	Detection and characterization of lung nodules, integration of imaging modalities for comprehensive assessment
Treatment Planning	Machine Learning, Predictive Analytics	Predicting treatment responses, guiding therapy selection, optimizing radiation therapy
Bronchoscopy	AI Algorithms, Robotic Assistance	Enhancing training and performance of bronchoscopists, safe navigation, reducing examination time and effort
Thoracic Surgery	Machine Learning, AI-Assisted Robotics	Preoperative evaluation, surgical planning, outcomes prediction, robotic assistance in surgical procedures

Bayesian network meta-analyses are a rapidly growing field of lung cancer research that has significant implications for all treatment methods. Traditional systematic meta-analysis seeks to combine clinical trial data to compare two therapies. However, in the contemporary management of lung cancer, it is often the case that there are more than two treatments available. In such situations, a typical meta-analysis needs to be revised. Network analysis offers two techniques to tackle this issue: an entirely indirect treatment comparison using a shared comparator or a mixed approach combining indirect and direct comparisons from accessible trials (15).

### Artificial intelligence in bronchoscopy

AI in bronchoscopy has significant potential to enhance the performance of bronchoscopists, particularly novice doctors. AI can help identify bronchial segments, crucial for practical bronchoscopy training. AI-assisted training improved novice bronchoscopists’ performance in identifying bronchial segments. An AI co-pilot bronchoscope robot has been developed to assist novice doctors in conducting lung examinations. This system uses a robotic arm and AI algorithms to guide the bronchoscope, reducing the need for human intervention and improving safety. AI can assist in steering the bronchoscope, ensuring safe navigation and reducing the risk of catheter damage to lung tissues. This is particularly important for novice doctors who may need more expertise. AI can help reduce the time and effort required for bronchoscopy examinations. By automating specific tasks, AI can free doctors to focus on more complex procedures. AI can help ensure the safety of bronchoscopy procedures by reducing the risk of misoperations and improving the accuracy of diagnoses.

Further studies are needed to evaluate the effectiveness of AI-assisted bronchoscopy in clinical settings and to assess its impact on patient outcomes. AI should be integrated with existing bronchoscopy systems to enhance their capabilities and improve the overall efficiency of the procedure. AI-assisted training programs should be developed to educate novice doctors on using AI in bronchoscopy and improve their overall performance (16, 17).

### Artificial intelligence in thoracic surgery

AI in thoracic surgery has shown promising results, including diagnosis, preoperative evaluation, surgical planning, and outcomes prediction. AI has been used to diagnose lung cancer by analysing CT scans and other imaging modalities. For example, AI can predict if pulmonary nodules are benign or malignant with 95% accuracy. AI-based technologies have shown promising results in preoperative risk assessment, effectively aiding the decision-making process and integrated risk scores. Machine learning algorithms have been used to predict the risk of significant complications and mortality after surgery, enhancing the precision and accuracy of surgical procedures (18). AI has been used to predict postoperative outcomes, such as weaning from ventilators after lung resection surgery (19). AI-assisted display based on surgical videos is a new surgical application that can be used for training. AI should be integrated with existing systems to enhance their capabilities and improve the overall efficiency of thoracic surgery. Incorporating AI in patient selection processes is an emerging area that promises to optimise surgical outcomes by identifying the most suitable candidates for specific thoracic procedures. AI algorithms can analyse vast amounts of clinical data, including patient demographics, comorbidities, genetic profiles, and imaging studies, to predict which patients will most benefit from surgery. By enhancing the accuracy of patient selection, AI can help reduce surgical risks and improve overall outcomes (20).

Further studies are needed to evaluate the effectiveness of AI-assisted thoracic surgery in clinical settings and to assess its impact on patient outcomes (19, 21). AI techniques such as machine learning, deep learning, and radiomics have demonstrated impressive ability in identifying and describing lung nodules. As a result, they contribute to precise lung cancer screening and diagnosis. These systems can analyse different types of medical imaging. They can accurately detect and identify abnormal nodules, enabling prompt intervention. AI models have shown potential in using biomarkers and tumour markers as additional screening tools, effectively improving the precision and accuracy of early detection. These models can accurately differentiate between benign and malignant lung nodules, aiding radiologists in making more precise and well-informed diagnostic determinations.

Moreover, AI algorithms can combine several imaging modalities and clinical data, resulting in a more thorough diagnostic evaluation. AI models can accurately forecast treatment outcomes and assist in choosing the most effective medications by analysing reliable data such as patient demographics, clinical history, and genetic profiles. These models have demonstrated significant effectiveness in accurately predicting the probability of response and recurrence after targeted medicines and optimising radiation therapy for patients with lung cancer. Integrating these artificial intelligence techniques into clinical practice can assist in promptly identifying and effectively treating lung cancer, thereby enhancing patient outcomes by reducing mortality and morbidity (13).

Another critical area of interest is AI’s role in preoperative planning, mainly through 3D reconstructions. Advanced AI-driven imaging techniques can create highly detailed 3D models of the thoracic cavity, allowing surgeons to plan procedures with unprecedented precision. These models can assist in identifying anatomical variations and potential challenges before surgery, thereby reducing operative times and improving surgical outcomes. Integrating AI with virtual and augmented reality platforms for surgical simulation and training further exemplifies its transformative potential in preoperative planning (22).

A neural network to forecast the cardio-respiratory outcomes after surgery in patients diagnosed with NSCLC achieved a sensitivity and specificity of 100% in properly categorising risks for mortality and postoperative morbidity in a test group of patients. AI can assist in forecasting the occurrence of illness and death, ultimately resulting in enhancements in treatment choice.

Nevertheless, there is potential for robotic systems to acquire autonomy to carry out specific repetitive activities. Hence, the integration of AI into accurate surgical procedures is likely viable and has the potential to enhance the overall quality. However, it is essential to note that the research was conducted on pigs and is not already a standard element of clinical practice. There are still substantial uncertainties regarding the legal, regulatory, and ethical aspects, and currently, there are no Food and Drugs Administration authorisations for autonomous surgery. Thus, the influence of AI in the domain of lung cancer is now restricted. Still, it can benefit in the future, particularly when surgeons encounter challenges related to intricate thoracic architecture (23).

AI applications in postoperative management are equally significant. For instance, AI-driven systems can monitor chest drainage, analysing patterns to detect complications such as air leaks or infections early on. This real-time monitoring can lead to prompt interventions, reducing the length of hospital stays and improving patient recovery. Additionally, AI can be employed to personalise the administration of postoperative pain medication by predicting patient responses based on genetic and pharmacological data. This personalised approach ensures optimal pain management while minimising the risk of opioid overuse or other adverse effects (24, 25).

## Conclusion

AI is revolutionising screening, diagnosis, and healthcare management by analysing unstructured big data and iteratively learning from it (**Table 2**). Integrating artificial intelligence into lung cancer management heralds a new era of precision medicine, offering transformative potential to revolutionise diagnosis, treatment, and surgical procedures. By leveraging AI’s unparalleled capabilities in data analysis and predictive modelling, healthcare providers can achieve unprecedented accuracy and efficiency. However, realising this potential requires a concerted effort to address ethical, regulatory, and practical challenges. As we stand on the brink of this exciting frontier, the question remains: are we ready to embrace the future of AI-driven healthcare? With continued innovation and collaboration, the promise of AI in lung cancer management is within our grasp, poised to deliver improved patient outcomes and redefine the landscape of modern medicine.

**Table 2:** Benefits and Challenges of Artificial Intelligence (AI) in Healthcare.

Aspect	Benefits	Challenges
Efficiency	Rapid analysis of large datasets, freeing up human resources for more complex tasks	Ensuring accuracy and reliability of AI outputs, integrating AI systems into existing workflows
Precision	Enhanced diagnostic accuracy, personalized treatment plans based on detailed patient data	Addressing biases in AI models, ensuring the fairness and equity of AI-driven decisions
Safety	Reduced risk of human error, improved procedural safety through AI assistance	Mitigating risks associated with AI errors, ensuring compliance with regulatory standards
Ethical Considerations	Potential to improve patient outcomes and healthcare accessibility	Privacy concerns, ethical implications of AI decisions, transparency in AI algorithms
